# Complete Genome Insights into *Lactococcus petauri* CF11 Isolated from a Healthy Human Gut Using Second- and Third-Generation Sequencing

**DOI:** 10.3389/fgene.2020.00119

**Published:** 2020-02-26

**Authors:** Yun-Jing Ou, Qiao-Qiao Ren, Shu-Ting Fang, Ji-Guo Wu, Yun-Xia Jiang, Yi-Ran Chen, Yi Zhong, De-Dong Wang, Guo-Xia Zhang

**Affiliations:** ^1^ Department of Environmental Health, Guangdong Provincial Key Laboratory of Tropical Disease Research, School of Public Health, Southern Medical University, Guangzhou, China; ^2^ Department of Water Hygiene, Guangzhou Center for Disease Control and Prevention, Guangzhou, China

**Keywords:** *Lactococcus petauri*, complete genome, insight, second-generation sequence, third-generation sequence

## Abstract

*Lactococcus petauri* CF11 was originally isolated from the gut of healthy humans. To determine the underlying molecular and genetic mechanisms of the probiotic potential of CF11, we performed complete genome sequencing, annotation, and comparative genome analysis. The complete genome of *L*. *petauri* CF11 comprised of 1,997,720 bp, with a DNA G+C content of 38.21 mol% containing 1982 protein coding genes and 16 rRNA operons. We found that 1206 genes (56.05%) were assigned a putative function using the gene ontology (GO) resource. The gene products of CF11 were primarily concentrated in molecular function and biological processes, such as catalysis, binding, metabolism, and cellular processes. Furthermore, 1,365 (68.87%) genes were assigned an illative function using COGs. CF11 proteins were associated with carbohydrate transport and metabolism, and amino acid transport and metabolism. This indicates that CF11 bacteria can perform active energy exchange. We classified 1,111 (56.05%) genes into six KEGG functional categories; fructose-bisphosphate aldolase and the phosphoenol pyruvate:phosphotransferase system (PTS), which are necessary in producing short-chain fatty acids (SCFAs), were excited in the carbohydrate metabolic pathway. This suggests that *L*. *petauri* CF11 produces SCFAs *via* glycolysis. The genomic island revealed that some regions contain fragments of antibiotic resistance and bacteriostatic genes. In addition, ANI analysis showed that *L*. *petauri* CF11 had the closest relationship with *L*. *petauri* 159469^T^, with an average nucleotide consistency of 98.03%. Taken together, the present study offers further insights into the functional and potential role of *L*. *petauri* CF11 in health care.

## Introduction

The intestinal tract is the largest microecosystem in the human body. It contains a significant number of intestinal microbes, which are known as the intestinal flora. The ecological community of commensal, symbiotic, and pathogenic microorganisms share space in the human gut. Intestinal flora plays an important role in human health and disease prevention because they provide nutrition and energy to the host by producing short chain fatty acids (SCFAs), vitamins, and amino acids (Nicholson et al., 2012). Intestinal flora is closely related to many physiological functions, such as immunity and metabolism, which play a significant role in host health (Lynch and Pedersen, 2016; Thaiss et al., 2016).

The genus *Lactococcus* is a genus of lactic acid bacteria, which are members of the family Streptococcaceae. Most members of this genus are very helpful for making fermented dairy products, including cheese, yogurt, and butter (Fusco et al., 2019). *Lactococcus* also exists in the human gut. It is found that *Lactococcus lactis* was present in the gastrointestinal tract of the infant on the first day of the life (Park et al., 2005). *Lactococcus petauri* has the closest relationship with *L. garvieae* and is a facultative anaerobic, non-motile, non-spore forming, Gram-positive cocci (Goodman et al., 2017). To date, *L. petauri* has been obtained in a facial abscess of a sugar glider alone, and no functional evidence was published. Microbial genomics can offer further understanding of functional gene characteristics, metabolic pathways of functional genes, and interactive mechanisms between regulatory factors. Therefore, deep understanding of the genome sequence of *L. petauri* strains was required.

Sequencing technology can truly reflect the genetic information of genomic DNA, providing important functional predictions. The Pacific Bio Sciences (Pac Bio) sequencing platform, which is a single molecular sequencing technology (Iso-Seq), offers significant improvements over current sequencing technologies because of its high throughput nature, fast speed, and longer reads (Qi et al., 2019). However, the error rate of Pac Bio is higher than in second-generation sequencing (SGS) technologies, leading to a reduced accuracy of assembly (Koren et al., 2012). Therefore, short Illumina reads from second-generation data are used to assist the correction of the long-read third-generation data to improve the accuracy of genome assembly without increasing the cost of sequencing. Following this, hybrid assembly is performed in whole-genome sequences (Rahman et al., 2013; Pootakham et al., 2017).

In this study, *L. petauri* CF11 was sequenced using the second- and third-generation sequencing technologies. The hybrid assembly genome was obtained based on short Illumina reads and long Pac Bio reads. These data offer a good foundation for the future research on genome function annotation, comparative genome analysis, and re-sequencing.

## Materials and Methods

### Isolation and Genomic Sequencing of Strain

Fresh fecal samples were gathered from the large intestines of four healthy persons, 0.1 g feces was suspended in 1.0 mL PBS buffer. 100 μL suspension was coated evenly on MRS solid medium. The fastest-growing single colony was named CF11. The strain was reserved at −80°C until next experiments.

This strain was inoculated in MRS solid medium at 37°C in anaerobic culture for 24 h. A single colony was harvested into 3 × 100 mL MRS liquid medium to create an enriched culture at 37 °C for 24 h. The thalli were harvested by centrifugation at 5000 rpm at 4°C for 10 min and then incubated in liquid nitrogen for 10 min. The total genomic DNA of CF11 was extracted and purified using a QIAGEN DNA Investigator Kit, according to the manufacturer's instructions. A whole genome shotgun strategy was used for sequencing. Briefly, we built a library with different inserts, and the whole genome sequences were obtained based on the Illumina MiSeq and real-time single molecule sequencing technology (Besser et al., 2018). The sequencing work was complete in Nextomics Biosciences Co., Ltd (Wuhan, China). Reads were assembled using Canu-SMART *de novo*, after quality control (Koren et al., 2017) and corrected using Pilon version 1.22 (Walker et al., 2014) combined with second-generation sequencing data. MECAT could also be used for fast mapping, error correction, and *de novo* assembly (Xiao et al., 2017). After the assembly, data were compared with the genome using Minimap2 (Li, 2018). The sequencing depth of each site was counted using SAMtools (Li et al., 2009). DNA modifications, such as 5 mC and 6 mA, could be detected by deep recurrent neural network on sequencing data (Xiao et al., 2018; Liu et al., 2019).

### Genome Annotation

The coding gene was predicted with prodigal (Hyatt et al., 2010) and the complete coding region was retained. Prediction results were integrated with their own scripts, and the locus tag numbers were assigned according to the gene sequence for subsequent analysis. In this experiment, tRNA genes were compared and predicted using tRNAscan-SE (Lowe and Chan, 2016). rRNA genes were predicted using RNAmmer (Lagesen et al., 2007). We used Infernal (Nawrocki and Eddy, 2013) to search the Rfam database (Kalvari et al., 2018) to compare and predict ncRNA. We found that >80% of the sequence length in the database was retained.

After extracting the encoded protein, Interroscan was used for annotation (Jones et al., 2014). We extracted the annotation information from TIGFAMs, Pham, and GO database (Haft et al., 2013). The GO annotation of coding genes in *L. petauri* CF11 was predicted using BLAST2 GO software. Pathway analyses were performed using the Genomes (KEGG) (Kanehisa et al., 2014) annotation service. The best results, with >30% coverage, were retained and mapped to the corresponding KEGG Pathway. Encoded proteins were compared with the COG database (Galperin et al., 2015) using rpsblast for COG functional annotation. Following this, protein coding genes that corresponded to COG functional numbers with the best consistency were selected. We categorized COG functional proteins according to the corresponding relationship between the number and classification directory. After the completion of both structural and functional annotation, we integrated the results to generate a final gff3 comment file. Next, tbl2asn was used to convert the comment and genome information into gb and sqn format files that were uploaded to NCBI directly. Sequencing depth, GC distribution, GC-skew, and genome structure were analysed using self-contained scripts. Finally, the ring map was drawn using Circos (Krzywinski et al., 2009).

### Phylogenetic Analysis of *L. petauri* CF11

The 16s rRNA sequence of *L. petauri* CF11 was compared with the NCBI database to discover strains similar to CF11. The 16s rRNA sequence phylogenetic tree of CF11 was constructed using MEGA X software (Kumar et al., 2018). The evolutionary relationship of the whole genome sequence was evaluated from different sources of strains using OrthoANI (https://www.ezbiocloud.net/tools/ani). The ANI values of the eight strains were calculated on the http://enve-omics.ce.gatech.edu/ani/index. DNA-DNA hybridization (DDH) was calculated using GGDC 2.1(http://ggdc.dsmz.de/ggdc.php).

## Results and Discussion

### General Genome Features of *L. petauri* CF11

There were 2,350,677,266 bp raw data outputs and 2,067,135,965 bp that underwent quality control in this study. And totally 217,153 reads were determined as valid with N50 of 12,313 bp. The longest reads was 98,214 bp. We obtained the genome of *L. petauri* CF11, which was comprised of 1 contig consisting of 1,997,720 bp, with an average G+C content of 38.21 mol% of the genome (**Table 1**). *L. petauri* CF11 has a small genome size and high G+C content relative to ten *Lactococcus* species/subspecies, where total genome size ranges from 1.99 (*L*. *plantarum*) to 2.46 Mb (*L. lactis* subsp. *lactis*). In addition, G+C content ranges from 34.81 (*L*. *lactis* subsp. *hordniae*) to 39.67 mol% (*L*. *raffinolactis*) (Yu et al., 2017). To date, one other study has reported the gene characterisation of *L*. *petauri* before this report. Furthermore, a genomic island was identified in the genome of *L. petauri* CF11, which was related to the bacterial fitness and virulence (Wu et al., 2014). We selected previously reported whole genome sequences for a comparative analysis, including contained *L*. *lactis* subsp. *lactis* ATCC 19435^T^, *L*. lactis subsp. *hordriae* LMG 8520^T^, *L*. *lactis* subsp. *cremoris* LMG 6897^T^, *L*. *petauri* 159469^T^, *L*. *garvieae* NBRC 100934^T^, *L*. *raffinolactis* NBRC 100932^T^, *L*. *chungangensis* DSM 22330^T^, and *L*. *plantarum* NBRC 100936^T^. *L*. *petauri* CF11 is composed of a complete chromosome and the gene content is similar to that of *L*. *plantarum* NBRC 100936^T^ and *L*. *garvieae* NBRC 100934^T^ (**Table 2**). *L. petauri* CF11 has a smaller genome size and lower number of predicated genes when compared with *L. petauri* 159469^T^. The isolated source is important parameter in assessing strain function. *L*. *garvieae* is as a pathogen found in diseased buffalos, cows, cats, dogs, and poultry. In contrast, *L*. *garvieae* isolated from healthy animals or the environment act as a probiotic (Vendrell et al., 2006). In this study, we obtained CF11 from the intestines of healthy individuals; therefore, its potential function as a probiotic should be investigated further.

**Table 1:** Basic genomic characteristics of *L. petauri* CF11.

Feature	Chromosome	% genome
Genome size (bp)	1,997,720	100
G + C content	763,344	38.21
5S rRNA genes	6	0.03
16S rRNA genes	5	0.38
23S rRNA genes	5	0.72
Open reading frames (ORFs)	1982	87.65
Genomic island	1	1.79

**Table 2:** Comparative genome features of strain CF11 and the most closely related strains.

Strain	Size（bp）	G+C mol%	Predicated gene number	Isolated source
*Lactococcus petauri* CF11	1997720	38.2	1982	Human intestines
*Lactococcus lactis* subsp. *lactis* ATCC 19435 ^T^	2514221	35.2	2665	Milk (dairy starter)
*Lactococcus lactis* subsp. *hordriae* LMG 8520 ^T^	2435575	34.8	2523	Leaf hopper (insect)
*Lactococcus lactis* subsp. *cremoris* LMG 6897 ^T^	2367195	35.5	2469	Cheese starter
*Lactococcus petauri* 159469 ^T^	2397176	37.7	2356	Facial abscess in a sugar glider
*Lactococcus garvieae* NBRC100934 ^T^	2028352	38.5	2054	Water buffalos
*Lactococcus raffinolactis* NBRC100932 ^T^	2179192	39.8	2157	Sour milk
*Lactococcus chungangensis* CAU28 ^T^	2214941	38.6	2185	Activated sludge
*Lactococcus plantarum* NBRC100936 ^T^	1977763	36.7	1896	Frozen pea

All data were created from NCBI GenBank nucleotide database except for strain CF11.

### Genome Annotation

**Figure 1:**
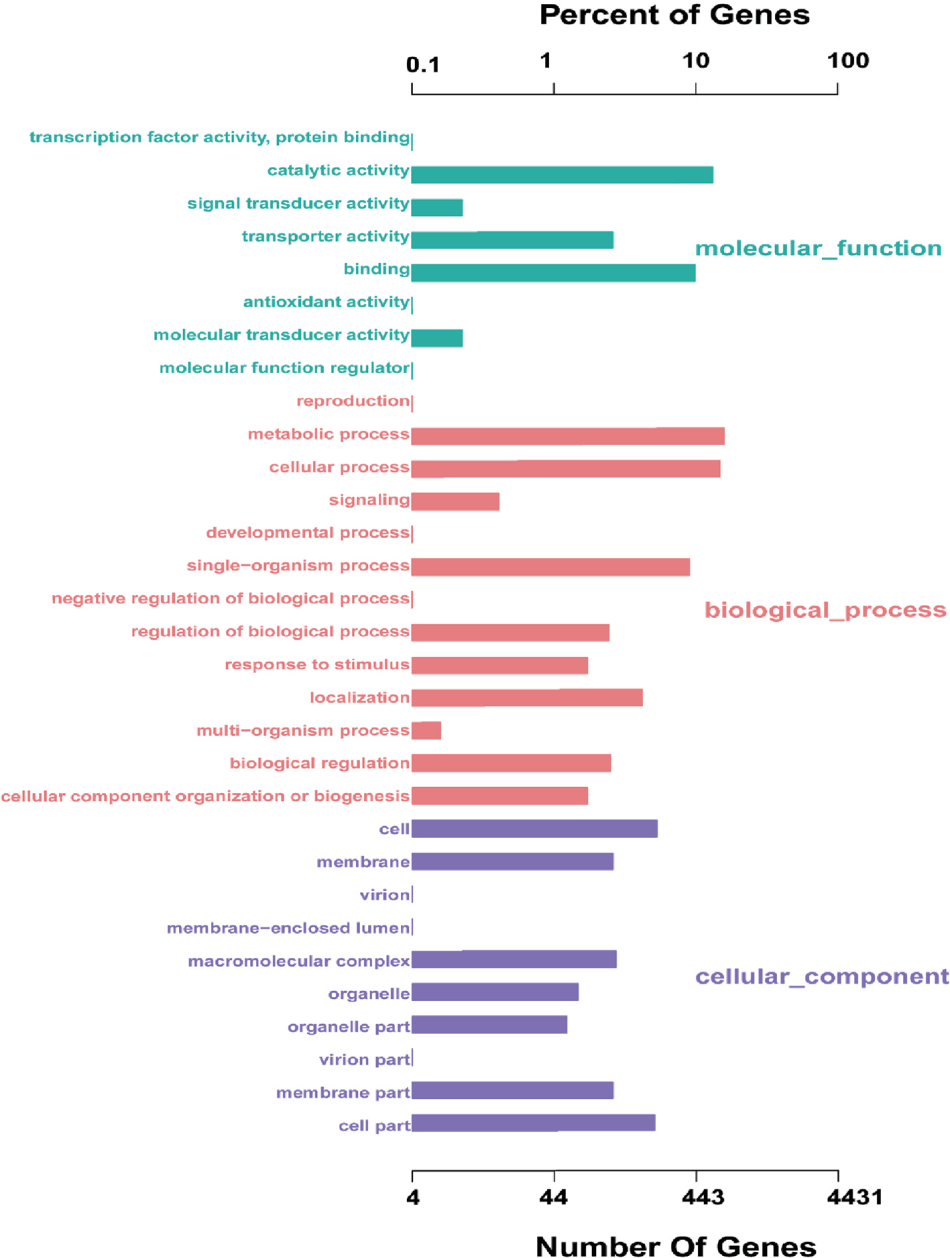
*L. petauri* CF11 genome-encoded protein GO functional annotation. The green, red, and purple bars represent the molecular functions, biological processes, and the cellular components of the genes, respectively.

The coding sequence of *L. petauri* CF11 was predicted, with a total of 1982 coding sequences (CDSs) in the identified genome. We assigned 1206 genes (56.05%) to a putative function by Gene Ontology (GO). Interestingly, 87.56%, 33.08%, and 40.29% of the genes encoded for molecular functions, cellular components, and biological processes, respectively (**Figure 1**). These results showed that the gene products of CF11 were linked with molecular functions, such as catalytic activity, binding, and transporter activity.

**Figure 2:**
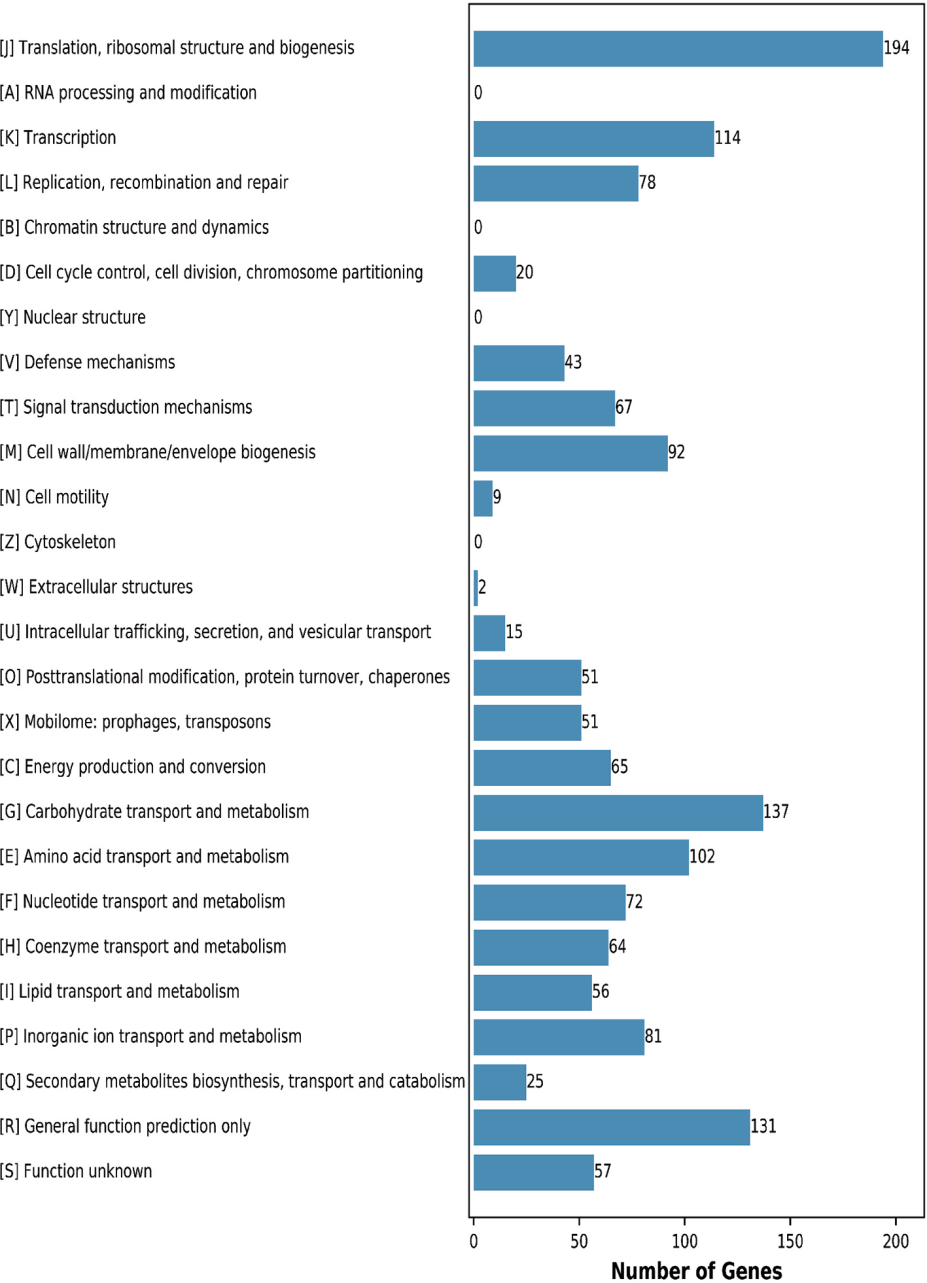
COG functional annotation of the genomic encoding proteins in *L. petauri* CF11. A–Z letters represent different COG functions. A: RNA processing and modification; B: Chromatin structure and dynamics; C: Energy production and conversion; D: Cell cycle control, cell division, and chromosome partitioning; E: Amino acid transport and metabolism; F: Nucleotide transport and metabolism; G: Carbohydrate transport and metabolism; H: Coenzyme transport and metabolism; I: Lipid transport and metabolism; J: Translation, ribosomal structure, and biogenesis; K: Transcription; L: Replication, recombination, and repair; M: Cell wall/membrane/envelope biogenesis; N: Cell motility; O: Posttranslational modification, protein turnover, chaperones; P: Inorganic ion transport and metabolism; Q: Secondary metabolites biosynthesis, transport, and catabolism; R: General function prediction; S: Function unknown.

This analysis assigned 1365 genes (68.87%) to an illative function by the Clusters of Orthologous Groups (COGs) database. The CF11 proteins were classified into functional categories for translation, ribosomal structure, and biogenesis (J,194 genes); carbohydrate transport and metabolism (G,137 genes); transcription (K, 114 genes); and amino acid transport and metabolism (E, 102 genes) (**Figure 2**). These regulators act on specific genes to control their expression and confer an advantage when present in the gut, which can assess the mechanisms employed to survive in this harsh environment (Zhang et al., 2019). We predicted that 9.60% of genes were associated with general functions, while 4.18% of the proteins were poorly characterized. Further analysis is required to elucidate their underlying mechanisms. In addition, 31.13% of the genes were not annotated by COG.

**Figure 3:**
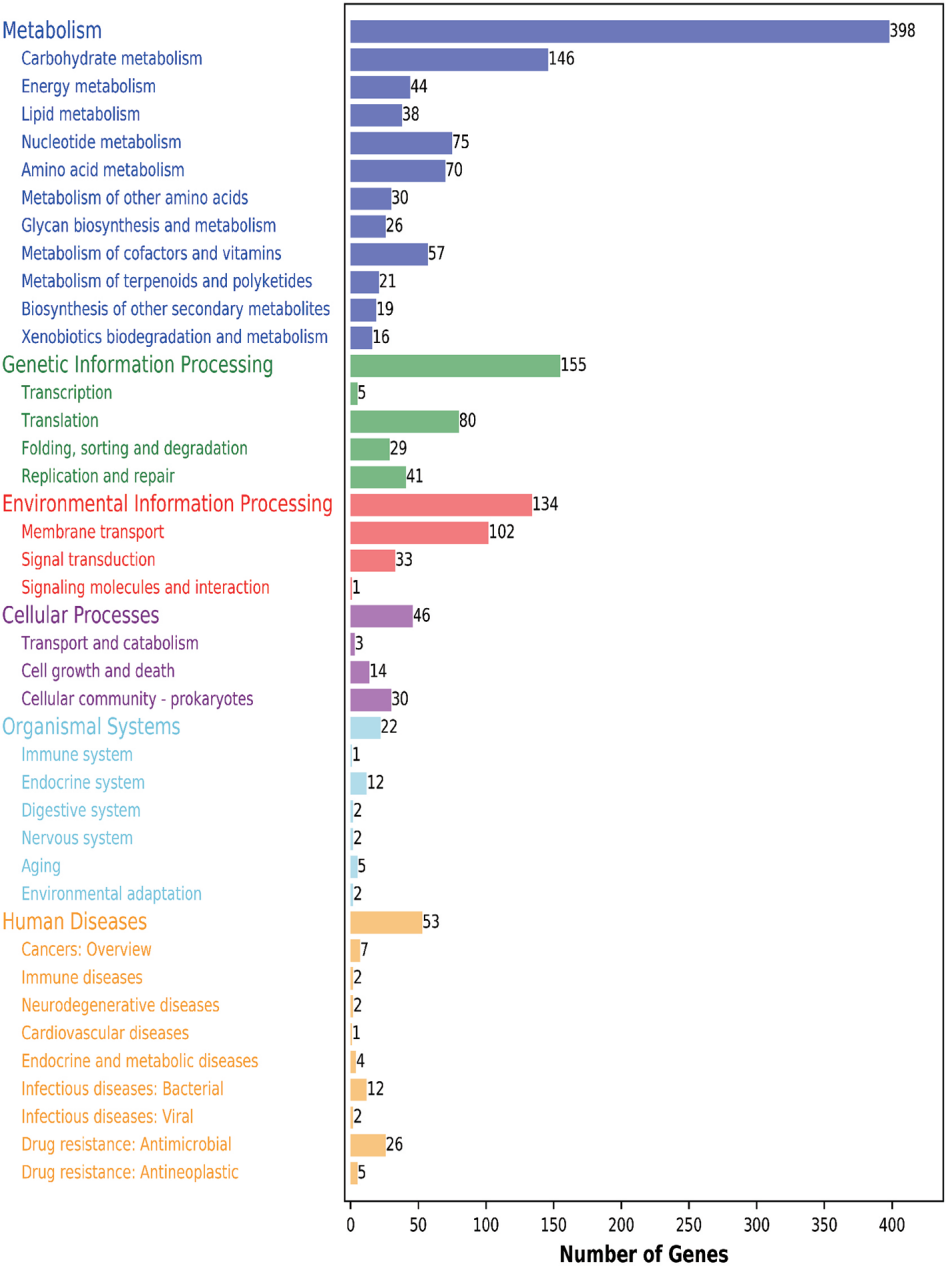
KEGG functional annotation of genome-encoding protein in *L. petauri* CF11. Blue: metabolism; Green: genetic information processing; Red: environmental information processing; Purple: cellular process; Light blue: biological system; Yellow: human disease.

**Figure 4:**
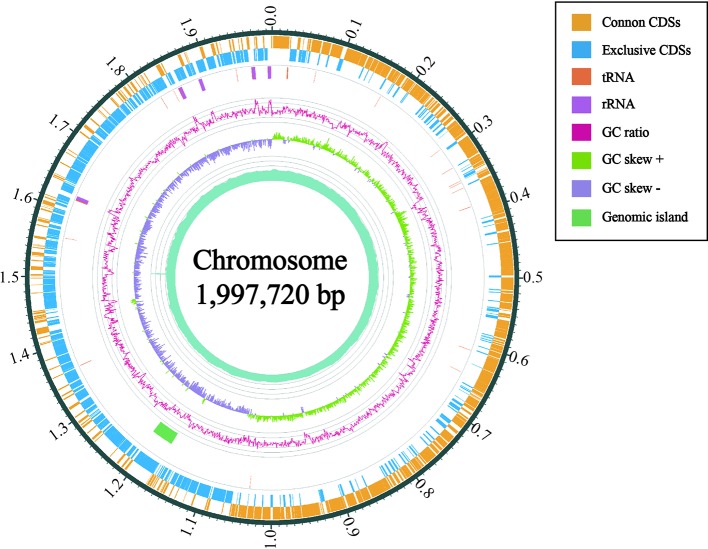
Circular map of the chromosome of *L. petauri* CF11. From outside to inside are the encoding genes (positive chains), encoding genes (negative chains), tRNA (orange) and rRNA (purple), genomic island (green), GC ratio (pink; mean GC is the reference line, the lines protruding outward and inward are above and below the mean, respectively; the GC-skew purple and green indicate <0 and >0, respectively.

Furthermore, 1111 genes (56.05%) were classified into six KEGG functional categories (**Figure 3**). These were primarily linked with metabolism (35.82%), genetic information processing (13.95%), environmental information processing (12.06%), cellular processes (4.14%), organismal systems (1.98%), and human diseases (4.77%). Each category contains its own metabolic processes. We found that 41 genes related to the viability of the bacteria were mapped to five KEGG pathways (phage replication initiation proteins; DNA replication proteins; replication initiation and membrane attachment proteins; ribosomal proteins; and the DNA replication and repair protein, RecF). This indicates that these genes play a vital role in these five pathways. In addition, we found that 11 out of 398 metabolic genes were associated with the pyruvate pathway, in which pyruvate metabolites are synthesized. Further, 13.14% of genes were linked to the carbohydrate transport metabolism. The gene coding for a key enzyme of glycolysis pathway, fructose-bisphosphate aldolase, exists in the genome containing all the genes that are required to degrade glucose to pyruvate. Pyruvate can be converted into lactic acid *via* the lactic dehydrogenase gene. Interestingly, several enzymes relating to pyruvate conversion, such as α-acetolactate synthetase, pyruvate-formate cleavage synthase, and lactate dehydrogenase, were confirmed in the genome of CF11 (**Figure 4**). The CF11 genome encoded 35 phosphoenol pyruvate-dependent PTS EII complexes related to the transport of carbon sources, including cellobiose, fructose, galactitol, lactose, mannose, sucrose, trehalose, mannitol, and maltose. The PTS is related to catalyze sugar transport as well as sugar phosphorylation (Saier, 2015). This suggests that L. petauri CF11 produces SCFAs *via* glycolysis.

### Phylogenetic Comparison of *L*. *petauri* CF11

**Figure 5:**
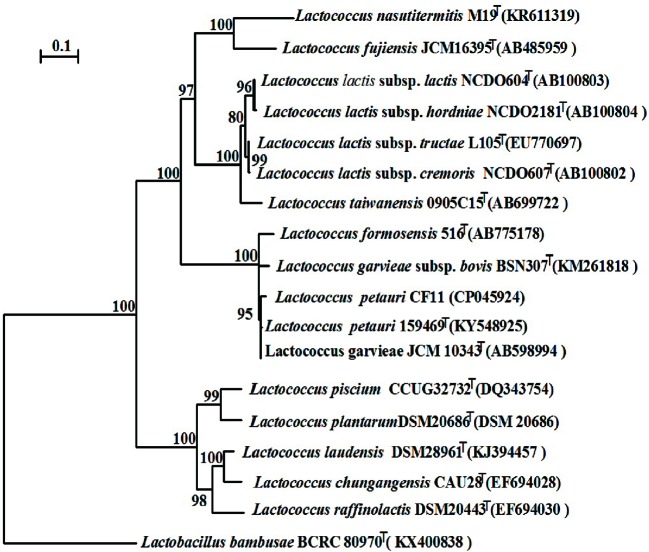
Neighbor-joining phylogenetic tree based on the 16S rRNA gene sequences of CF11 and closely related species within the genus *Lactococcus*. Bootstrap values (>70%) based on 1000 replications are listed as percentages at the branching points. The bar represents 0.1 substitutions per nucleotide position. *Lactobacillus banbusae* BCRC 80970^T^ is presented as the outgroup.

We constructed a phylogenetic tree based on the 16S rRNA gene sequence. The most closely related trains were *L. garvieae* NBRC 100934^T^ and *L*. *petauri* 159469^T^, with similarities above 99.7% and 99.5%, respectively (**Figure 5**). The 16S rRNA gene similarities were significantly higher than the proposed species delimitation threshold 98.65% (Kim et al., 2014). But we can’t confirm the strain CF11 should be *L. garvieae* or *L*. *petauri*. So, the genomic analysis must be performed. *L*. *petauri* CF11 had the closest relationship with *L*. *petauri* 159469^T^, with OrthoANIu and ANI values of 98.04% and 98.02%, respectively, at the genome level (**Table 3**). An ANI value of 97% is a species threshold; therefore, *L*. *petauri* CF11 and *L*. *petauri* 159469^T^ may be same species (Richter and Rosselló-Móra, 2009). The highest DDH value (DDH = 81.80%) was obtained for the isolates, *L*. *petauri* CF11 and *L*. *petauri* 159469^T^. The gold standard threshold for species boundaries is a DDH of 70% similarity (Meier-Kolthoff et al., 2013). This offers further confirmation that CF11 belongs to the already described species, *L. petauri*. The other cluster contained seven isolates with ANI and DDH values of 77–94% and 22–55%, respectively. The 16S rRNA gene phylogenetic result cannot be used as an evaluation indicator alone for strain taxonomic position, the more gene or genomic level comparation is very essential.

**Table 3:** Results of ANI calculations and in silico DDH (*is*DDH) of the strain CF11 compared with the several related species.

Strains	Ortho ANIu (%)	ANI (%)	isDDH (%)
*Lactococcus lactis* subsp. *lactis* ATCC19435^T^	72.69	78.88	22.70
*Lactococcus lactis* subsp. *hordriae* LMG8520 ^T^	72.39	78.92	22.70
*Lactococcus lactis* subsp. *cremoris* LMG6897 ^T^	72.76	79.18	24.60
*Lactococcus petauri* 159469 ^T^	98.04	98.02	81.80
*Lactococcus garvieae* NBRC100934 ^T^	93.61	93.27	54.30
*Lactococcus raffinolactis* NBRC100932 ^T^	68.93	77.88	24.10
*Lactococcus chungangensis* DSM22330 ^T^	69.18	77.39	24.90
*Lactococcus plantarum* NBRC100936 ^T^	69.11	77.48	22.80

## Data Availability Statement

The datasets generated for this study can be found in GenBank. The Whole-genome sequence accession No: CP045924.

## Ethics Statement

This study was approved by the Ethical Committee of Southern Medical University, Guangzhou. The participants provided their written informed consent to participate when providing stool samples.

## Author Contributions

S-TF and G-XZ conceived and designed the experiments. S-TF, Q-QR, J-GW, and Y-XJ carried out the experiment of this study. S-TF, Y-JO, G-XZ, Y-RC, YZ, and D-DW participated and analyzed data in the experiment. Y-JO and G-XZ prepared the manuscript. All authors have read and approved the manuscript in its final form.

## Funding

This work was supported by the National Science Foundation of China (NSFC 31500076), Science and Technology Program of Guangzhou, China (201904010161) and Guangzhou Municipal Science and Technology Project (20191A011063).

## Conflict of Interest

The authors declare that the research was conducted in the absence of any commercial or financial relationships that could be construed as a potential conflict of interest.
